# Enabling visibility of the clinician-scientists’ knowledge broker role: a participatory design research in the Dutch nursing-home sector

**DOI:** 10.1186/s12961-021-00715-z

**Published:** 2021-04-07

**Authors:** Margot Barry, Wietske Kuijer, Anke Persoon, Loek Nieuwenhuis, Nynke Scherpbier

**Affiliations:** 1 Department of Occupational Therapy at the HAN University of Applied Sciences, Kapittelweg 33, 6525EN Nijmegen, The Netherlands; 2 Faculty of Educational Science, Open University, Heerlen, The Netherlands; 3 Research Department of Public Affairs, HAN University of Applied Sciences, Nijmegen, The Netherlands; 4 Department of Research on Learning and Education, Radboud University Medical Centre Health Academy, Nijmegen, The Netherlands; 5 Department of Primary and Community Care, Radboud Institute for Health Sciences, Radboud University Medical Centre, Nijmegen, The Netherlands; 6 Faculty of Education at HAN University of Applied Sciences, Nijmegen, The Netherlands; 7 Department of Primary and Community Care, Radboud University Medical Centre, Nijmegen, The Netherlands

**Keywords:** Knowledge broker, Clinician-Scientist, Design research

## Abstract

**Background:**

A group of clinician-scientists and managers working within a Dutch academic network, experienced difficulties in clearly defining the knowledge broker role of the clinician-scientists. They found no role clarity in literature, nor did they find tools or methods suitable for clinician-scientists. Clarifying role expectations and providing accountability for funding these knowledge broker positions was difficult. The aim of this research was to design a theory-informed tool that allowed clinician-scientists to make their knowledge broker role visible.

**Methods:**

A participatory design research was conducted in three phases, over a 21-month period, with a design group consisting of an external independent researcher, clinician-scientists and their managers from within the academic network. Phase 1 constituted a literature review, a context analysis and a needs analysis. Phase 2 constituted the design and development of a suitable tool and phase 3 was an evaluation of the tool’s perceived usefulness. Throughout the research process, the researcher logged the theoretic basis for all design decisions.

**Results:**

The clinician-scientist’s knowledge broker role is a knowledge-intensive role and work-tasks associated with this role are not automatically visible (phase 1). A tool (the SP-tool) was developed in Microsoft Excel. This allowed clinician-scientists to log their knowledge broker activities as distinct from their clinical work and research related activities (phase 2). The SP-tool contributed to the clinician-scientists’ ability to make their knowledge broker role visible to themselves and their stakeholders (phase 3). The theoretic contribution of the design research is a conceptual model of professionalisation of the clinician-scientist’s knowledge broker role. This model presents the relationship between work visibility and the clarification of functions of the knowledge broker role. In the professionalisation of knowledge-intensive work, visibility contributes to the definition of clinician-scientists broker functions, which is an element necessary for the professionalisation of an occupation.

**Conclusions:**

The SP-tool that was developed in this research, contributes to creating work visibility of the clinician-scientists’ knowledge broker role. Further research using the SP-tool could establish a clearer description of the knowledge broker role at the day-to-day professional level and improved ability to support this role within organisations.

## Background

The value of clinician-scientists (CSs) in linking the often disparate contexts of research and clinical practice, by virtue of having professional jurisdiction in both, is uncontested in the discourse of translational research [1]. As knowledge brokers, CSs have the advantage of habitual, first-hand experience of clinical work and research processes. This facilitates strategic networking [2] and the involvement of stakeholders [3, 4] in designing and executing broker activities that link research and practice [5]. Thompson and Schwartz Barcott [6] define a knowledge broker as “one who connects science and society by building networks and facilitating opportunities among knowledge producers and knowledge users”. A CSs operational involvement in both research and clinical practice, facilitates unique insights into the goals, priorities and organisational logics of both contexts [7]. CSs are in a position to display sensitivity and responsiveness to both contexts [4]. This strengthens CSs ability to design tenable knowledge broker activities that balance scientific and economic interests and consequently have a higher potential to appeal to both scientists and care providers [5]. Activities carried out as part of the knowledge broker role can be categorised under three components: forming and sustaining partnerships; facilitating knowledge application; and creating new knowledge [6].

Bornbaum et al. [8] conceptualise the three components of the knowledge broker role as knowledge management, linkage and exchange, and capacity building [8].

As the definition and the components above suggest: the knowledge broker role of CSs is distinct from the clinical- and research roles [5] and it requires competencies additional to those required to execute clinical- and research tasks [5]. Successful knowledge brokering by CSs leads to an increased volume of clinically relevant research results [2, 9, 10] and increased evidence application in practice [11, 12]. The nature of the connection brokered by CSs is ideally bilateral and dynamic in nature, whereby the research context and the clinical context inform each other [7].

Despite the espoused value of the CS as a knowledge broker, little published information exists about the exact nature of this role at the day-to-day professional level. The connection between research and practice is frequently assumed to occur by virtue of the CSs professional jurisdiction in both settings [5]. The knowledge broker role of CSs is not yet concrete enough to constitute a professionalisable work package that lends itself to the establishment of exclusive professional jurisdiction [1, 13]. This may in part emanate from previous limited clarity on the definition of the knowledge broker role in healthcare disciplines. A clear definition and conceptual model was published recently, in 2019 [6] to guide nurse-scientists in the establishment of their knowledge broker role. To date, however, many CSs still occupy an ambiguous intermediary position between research and practice [14], they struggle with professional identity issues and workload across disparate roles [1, 14]. Their research and clinical activities are visible, easily quantified and thus contributing to the establishment of subject matter expertise or specialisation. Their broker activities, however, remain unclear and potentially unseen, whilst these activities consume a substantial portion of time. Some broker activities are viewed as logistic and generic tasks that do not bestow professional expertise and hence do not require professional development effort in their own right [15], for example implementation facilitation [4]. CSs unsurprisingly experience workload difficulties and CS numbers are declining [14]. The aim of this research was to design a theory-informed tool that allowed clinician-scientists to make their knowledge broker role visible.

### Context of this research

This research was conducted in a Dutch academic network: a collaboration between fifteen nursing-homes and an academic medical research institute. As part of the strategy to link research and clinical practice, the academic network employed twelve master-educated CSs in 2018 and 2019. The CSs were tasked with catalysing both care-informed research and evidence-informed implementation initiatives. The managers of the CSs voiced concerns about their limited ability to demonstrate accountability for funding these broker positions. The CSs reported insecurities about role-expectations and difficulties in making their broker role visible. The difficulty in clearly defining the CSs broker role presented a practical challenge in this academic network. The managers and CSs were unable to find a tool suitable for making the CS broker role visible. In response to this practical difficulty, we conducted a participatory design research aimed at developing a practical method of making the broker role of the CSs visible.

## Methods

Design research is a genre of research that is collaborative and suitable for the design and construction of tools that are required to solve practical problems [16]. It contributes to existing theory [16], in this case, the theory on the visibility and professionalisation of the CS broker role. Design research attempts to balance research rigour with practical relevance.

**Fig. 1:**
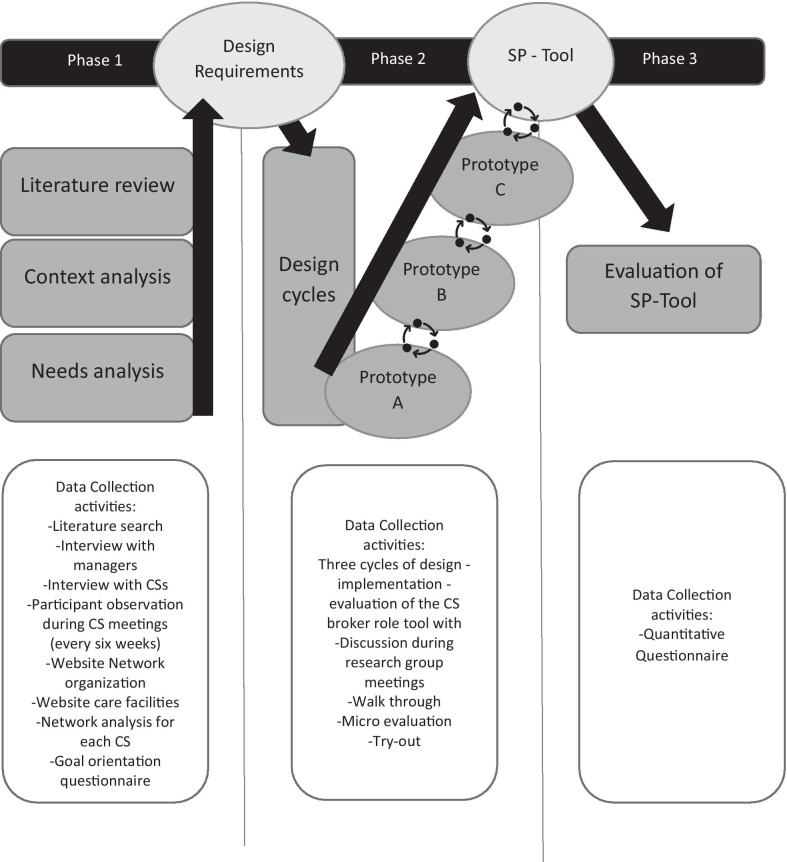
Schematic representation of the design research phases with data collection activities at each phase

We invited all CSs and managers from within the academic network to form a design-group together with an external independent researcher (MB), with the aim of designing a tool that allows CSs to make their broker role visible. We implemented the three phases of design research [16]. Phase 1, the orientation phase, consisted of a literature review, a context analysis and a needs analysis to explore the needs of the academic network, in order to draft a design requirements and a design proposition [16]. Phase 2 consisted of the cyclical process of design, construction and evaluation of a broker-activity logging tool, that fulfilled the design requirements drafted in phase 1. Phase 3 entailed evaluation of the final tool designed. These three phases and the concomitant data collection activities are summarised in Fig. 1.

The literature review in phase 1 was conducted from May to August 2017 according to the realist review method and was published separately [5]. The literature review served as a starting point from which the design-group worked collaboratively during the remainder of the project, which entailed six-weekly meetings from July 2018 to July 2019. The final evaluation extended to March 2020.

In Phases 1 and 2, data were collected during design-group meetings, individual interviews with CSs, participant observation of supervision meetings between CSs and their manager, questionnaires, walkthroughs, micro-evaluation of elements of the designed tool and try-outs of the tool. Data were in the form of minutes of meetings, sociograms, notes in the researchers logbook and member checked notes taken during interviews and conversations. Key assertions by participants were documented verbatim and member checked. In phase 3 an anonymous online questionnaire was distributed to CSs. They rated their perception of the tool’s effect on their ability to make their broker role visible, on a seven-point Likert scale ranging from ‘a lot worse’ to ‘a lot better', compared to a situation without the tool. CSs also indicated on a multiple-choice question how they planned to use the tool. An individual discussion and reflection was conducted with the manager after the final try-out of the tool.

We analysed data collected in phase 1 using framework analysis [17], which is suitable for research that develops new plans and actions. The independent researcher analyzed the raw textual data following the steps of coding, indexing, charting, mapping and interpretation [17] using a framework in Microsoft Excel. The codes comprised the inner- and outer context factors necessary for effective CS brokerage as identified in the literature review [5]. To enhance credibility, a critical friend and co-author (AP) read the populated framework, to determine whether she agreed with the categorization of quotes under the existing codes. The interpretations arising from the framework analysis were shared and discussed with the entire design group.

During phase 2 (design and construction), data were collaboratively translated into changes required for the prototypes of the tool during design-group meetings. The CSs and the managers made practical suggestions. The answers from the online evaluation questionnaire in phase 3 were numerically tallied and reported in raw numbers.

Throughout all three phases, the researcher compared group discussion topics to available literature on the same topics. This assisted the researcher in interpreting the conversations through a theoretic lens and led to drafting, discussing, re-drafting and finalising the conceptual model.

### Reflexivity and authors’ positionality

The first author (M.B), an occupational therapy lecturer, was an independent researcher from an external academic institute. She planned and executed this research in collaboration with the design group. M.B was not a colleague of the CSs nor their managers and did not know them prior to the commencement of this research. M.B used a researcher’s logbook to capture her observations and assumptions throughout the research process. She shared her assumptions with the design group in order to maintain a focus on their practical interest in this research process. The third author (A.P) was a CS and a member of the design group, who’s contribution to the writing process, extended the participatory character of this design research into the writing of this article. The remaining authors (W.K, senior researcher, L.N, professor of education and N.S, general practitioner and associate professor) were senior researchers who provided consultancy and assistance to the independent researcher in planning and executing the research from the proposal stage to the writing of this article. They had no contact with participants of the design group.

## Results

The design-group consisted of eleven of the twelve CSs and their manager. One CS did not consent to participation. All participants gave written informed consent to participate in the research. The professional backgrounds of the CSs were in nursing, physiotherapy, speech and language therapy, sport-and-movement therapy and psychology. One CS fulfilled a part-time management role within the network. Nine of the eleven CSs were clinical professionals within care facilities, who received funding by the academic network to dedicate one day per week to the CS broker role. Two were researchers employed by the academic research institute, who received funding to spend one day per week as a CS within an assigned care facility.

### Phase 1

The context and needs analysis showed that the CS role was new within this academic network and neither CSs nor managers knew exactly what to expect from this role. CSs experienced time pressure and felt that the number of requests they received for engagement always exceeded the time they had available. They reported that many requests did not fit the scope of their CS role and felt that their colleagues did not have a clear understanding of the CS role. CSs further reported uncertainty in their ability to prioritize requests and acknowledged that their choices hinged upon organizational priorities and their own skill profile. Phase 1 resulted in a design proposition: ‘to design a performance appraisal tool that makes the broker role of CSs visible’. The six collaboratively formulated design requirements for this tool were as follows: 1—data entry is not time consuming; 2—work in progress and work completed are explicated; 3—an individual profile of the CS is presented; 4—organizational barriers and facilitators to goal achievement are presented; 5—broker activities are linked to the organization’s priorities; and 6—the manner in which the CS is a catalyst in linking research and practice is shown.

### Phase 2

**Fig. 2:**
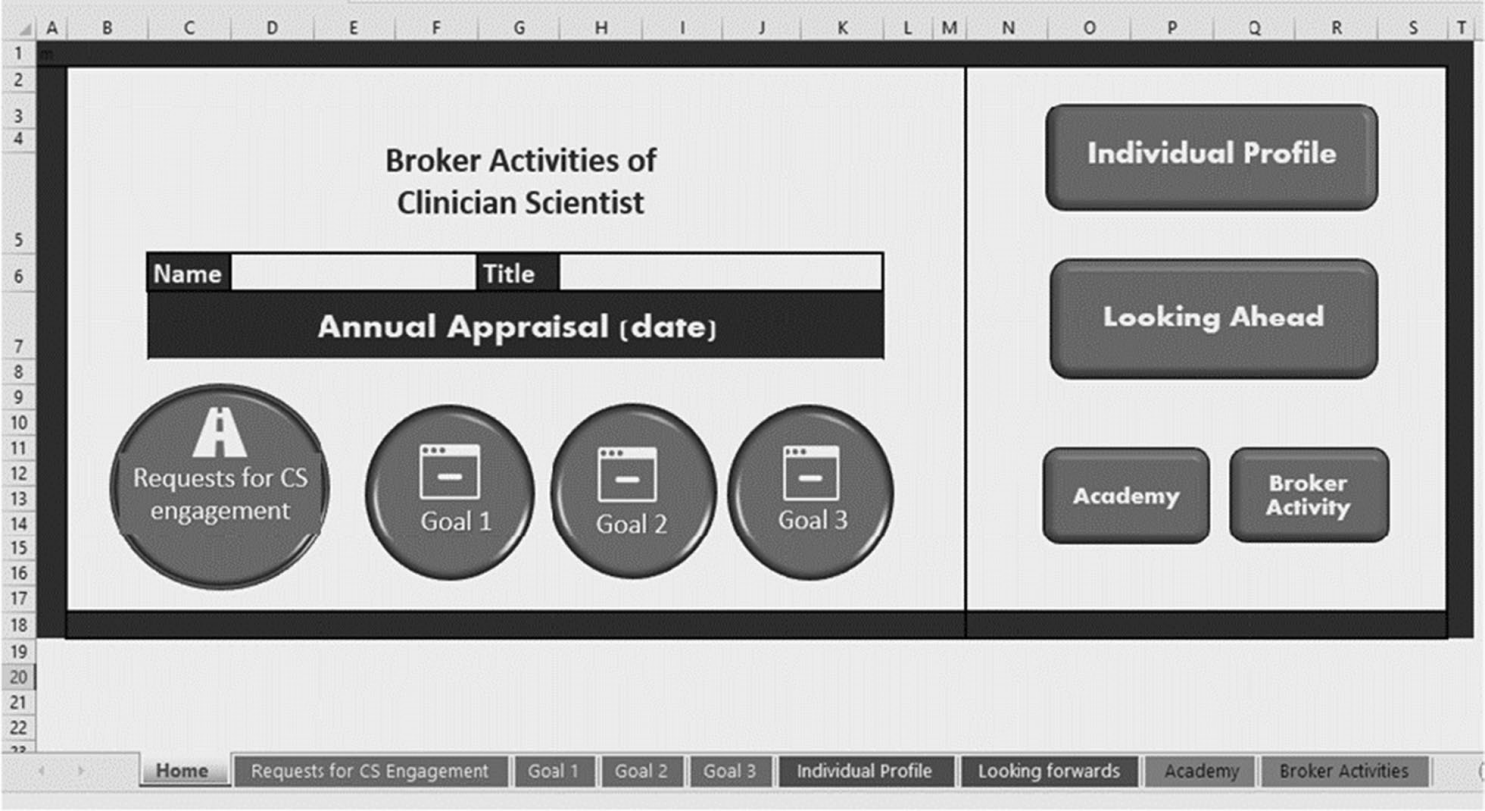
A screengrab from the navigation interface of the SP-tool: buttons link to pre-formatted spread sheets on which clinician-scientists can log relevant information

The practical result of the three design cycles of phase 2 was a tool in fulfilment of the above design proposition and the requirements. We called the final product, the Science-Practitioner tool (SP-tool) (see Fig. 2). The SP-tool was the result of three design cycles and made it possible for CSs to document their broker activities flexibly.

In the first design cycle, a narrative report format with pre-determined headings and sub-headings was designed and used. The report formed the basis for discussion during the CSs performance appraisal with their manager. This format was not deemed suitable by CSs and managers alike as it was static in nature. The managers could not ascertain common elements between reports in order to gain an understanding of the broker role independent of context. One manager (participant 1) said, “*We want to be able to assess whether the CSs are doing enough but we don’t know what ‘enough’ is*.” The CSs found that documenting their goals for the broker role on a Goal Attainment Scale [18] was too rigid and too specific. It did not assist in showcasing flexibility and sensitivity to the context. “*I need to show the activities I do within the broader goals of the CS function”* (participant 10). CSs felt it important to showcase all the requests they received and their subsequent decisions in prioritizing certain requests over others. *“I receive a tsunami of questions, but many are not suitable for a CS, more so for a researcher. I want to communicate more about my (CS) function.”* (participant 8). The report did not make the dynamic nature of the CSs network activity and social capital visible. Respondent 11 said, *“I link people in my network with each other and great things happen, but this is not visible anywhere”*.

Elements of the report that were seen as positive were that CSs could present their professional profile, the organizational barriers and facilitators to goal achievement and the categorization of the broker activities they executed in a theory informed framework of knowledge broker roles[8].

To address the difficulties, the second and third design prototype were MS Excel spreadsheets, which allowed all requests received by CSs to be documented, not only those that were accepted and operationalized by CSs. For each request logged, the CS could input data from dropdown lists about the nature of the request, the network partner involved in submitting the request, the complexity of work involved in operationalizing this, the relationship to the strategic goals of the organization and the nature of the broker activity required to address the request. Table 1 gives an example of entries made by CSs. The goals of the CS broker role were formulated more broadly and were able to accommodate a broad spectrum of requests from stakeholders.

**Table 1:** Data logged by clinician-scientists and mechanism from the literature review prompting its inclusion

Data requested on SP-tool	Example 1	Example 2	Example 3	Mechanism for effective execution of the broker role from the literature review (Barry et al. [5]) that prompted inclusion
Description of nature of request or question for CS engagement	Set up an on-site triage facility	Determine best use of available wearable hip injury prevention airbag belts in care home	How can we best prevent sarcopenia on the ABC ward?	Sensitivity and responsiveness to local context
Who submitted the question/assignment? Choose from the dropdown list: Clinician Researcher Funder Carer Patient Manager Other: Specify	Other: CS identified necessity this during clinical task performance and collaboration with medical team and management	Occupational Therapist	Dietician	Strengthening network relationships between researchers, clinicians, and policymakers; involvement of clinical staff in research; strategic focus on networking activities; involvement and enablement of key stakeholders
Nature of the question Research question Practice question Implementation question Other	Implementation question	Practice Question	Research question	Leadership opportunities in implementing research results in practice; balancing economic and scientific interests; direct involvement in key decision making in evidence-based change projects
Type of broker activity according to Bornbaum [8] required to operationalise the question Networking Knowledge management Capacity building A combination of the above None of the above	Capacity development	A combination of the above	Knowledge management	Focus on translation and practical applicability of research results; catalysing the formulation and conduct of care-informed research
Alignment with strategic focus of organisation Yes No Not sure	Yes	Yes	Yes	Alignment of own goals or values with organizational goals; ability to prioritize diverse work tasks across contexts

In contrast with the first prototype, the reporting structure in MS Excel secured an element of uniformity in reporting. This assisted managers in their understanding of the broker role. It allowed visibility of the conceptualisation which the stakeholders (network) have of the CS broker role, as seen in the type of requests for engagement. It also presents the conceptualisation which the CS has of their own role by showing which activities he/she prioritises and why.

### Phase 3

Seven of the eleven CSs completed the online questionnaire about the perceived usefulness of the SP-tool. They anticipated it as useful in making their tasks visible to stakeholders and improving their own understanding of the broker role. Five (*n* = 5; 71%) indicated that owing to the SP-tool, their ability to explicate their broker role was ‘much better’ and two (*n* = 2; 29%) indicated that it was ‘somewhat better’. None of the respondents chose the options indicating that their ability to explicate their broker role had remained the same or had become worse. The respondents’ intended use of the SP tool is shown in Table 2, which also reflects all the options on the multiple-choice question which they were posed.

**Table 2:** CSs intended use of the information obtained from the SP-tool

Intended use of the information obtained from the SP-tool	CSs responses Count (%)
For professional development purposes	2 (29)
As a basis for discussion of my functioning as a CS	3 (43)
To obtain insight into the questions from the clinical practice environment	5 (71)
For accountability purposes	7 (100)
To prioritise my own work tasks	3 (43)
Other	1 (14)

The CS selecting the option ‘other’ added that the information from the SP-tool would be used to showcase all activities, which the CS does on a day to day basis.

The manager implemented the SP-tool for use by all CSs in the next performance appraisal meeting of each CS.

## Discussion

The findings of this research resonate with previous research findings on several issues relating to the CS knowledge broker role. These include the time pressure experienced by CS as knowledge brokers [14], the difficulties with role clarity and professional identity of the CS [1, 14], the contextual specificity of the CS knowledge broker role [5] and the importance of networking and relationship management [6, 8]. This research adds the dimension of visibility of work to the discussion on topics relating to the CS knowledge broker role.

This design research has been useful in developing a practical theory-informed tool (the SP-tool), which allows individual CSs to make their knowledge broker role in a nursing-home context visible. Visibility of knowledge-intensive work is not automatic but intentionally constructed [19]. The SP-tool assists with intentional construction as it allows CSs to make broker activities, that belong neither exclusively to the jurisdiction of research nor that of clinical practice, structurally visible during performance appraisal.

**Fig. 3:**
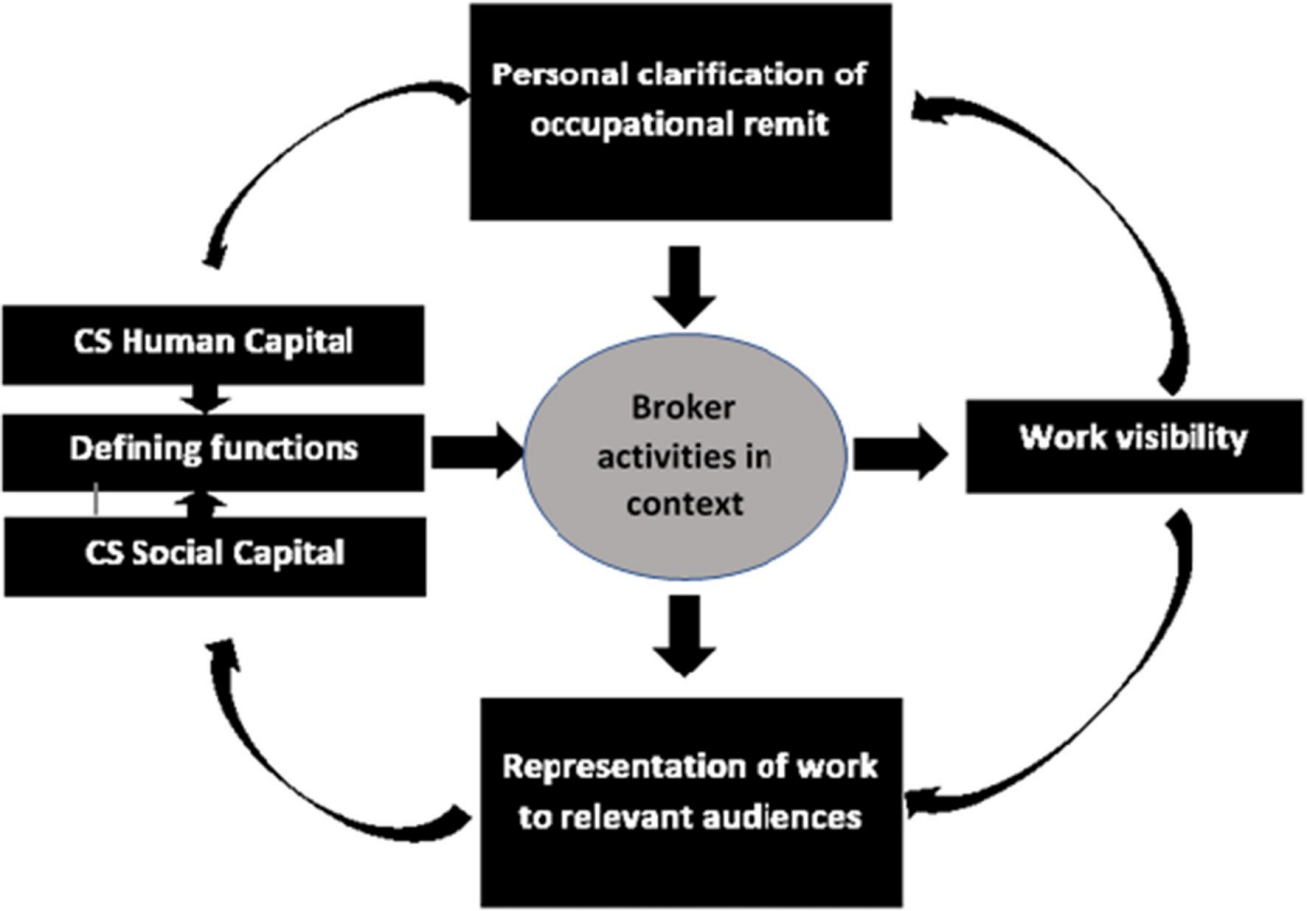
Conceptual model of professionalisation of the knowledge broker role of the clinician-scientists

Strategic construction of work visibility based on active engagement in an organisation, has been found to contribute to the professionalisation of various knowledge-intensive occupations in the corporate world [20]. This dynamic form of professionalisation stands in contrast to that of traditional healthcare professions, in which professionals are socialised to work according to a clearly defined and visible scope of practice. Professionalisation through engagement in a dynamic context, where the expectations and views of stakeholders inform the development of an occupational role is a process that might be new and unknown to CSs who stem from an academic health professions background. To illustrate this process, we contribute a conceptual model based on the findings of this design research. The model integrates work visibility with theoretic tenets of professionalisation for the CS broker role. A core element of professionalism is the existence of clearly defined work functions [21] and a shared understanding of these functions between the professional and the service-user. In our model (see Fig. 3), engagement with employers and service users, is a means to defining professional remit. This is in line with mechanisms described in less conventional professionalisation processes in other knowledge-intensive industries such as project management and design [20, 22]. A definition of functions constituting the occupational role of the CS is postulated through dynamic interaction in the work context. The definition of functions is not pre-determined by a state regulated monopoly such as is the case for medical professionals. We would like to propose this conceptual model for professionalisation of the CS broker role. Figure 3 presents the conceptual model.

Professionalisation is said to improve the reputation of a profession [23]. Interestingly, CSs are generally viewed positively and deemed important [5], despite their difficulties with role clarity.

In addition to its contribution to professionalisation, the SP-tool can be viewed as a boundary object [24], contributing to a common understanding between the CS and their manager. A collective understanding of professional role contributes to a collective identity of a profession [21] and might contribute to overcoming professional identity difficulties associated with the CS broker role.

A limitation of this research is the small number of participants, however, in the academic network all but one CS participated. Their contribution significantly enhanced the practical applicability of the SP-tool. A further known limitation of design research is its context specific nature and very limited generalisability. However, the clearly defined design proposition and requirements in this research might be of interest to CSs and managers in other contexts. The SP-tool and the proposed conceptual framework could be of interest in future more fundamental research into the day-to-day professional level of CSs. Future research using the SP-tool could provide a visible array of work activities associated with the knowledge broker role, and processes applied to prioritise the requests which CSs receive. These insights could assist in describing the day-to-day professional activity of the CS knowledge broker, to underpin its known strategic value and contribute to literature on this topic. Moreover, knowledge on the practical requirements for the CS knowledge broker role, facilitates the development of more structured training or mentoring processes to support CSs. At an organisational level, future research could provide insight into the outcomes achieved by CSs in relation to the strategic goals of the organisation. This potentially allows for better positioning of this role within an organisation, embedding it in existing structures, processes and communities.

## Conclusion

We developed the SP-tool, which contributes to creating work visibility of the CSs knowledge broker role, which is a knowledge-intensive role and work-tasks associated with this role are not automatically visible. Visibility might assist in the clarification of CS broker functions at the day-to-day professional level and contribute to professionalisation and improved support for CSs.

## Data Availability

The datasets used and/or analysed during the current study are available from the corresponding author on reasonable request. This includes the Science-Practitioner Tool which was designed during this research.

## Abbreviations

CS: Clinician Scientist
SP-tool: Science-Practitioner tool

## References

[1] Hendriks B, Simons A, Reinhart M (2019). What are clinician scientists expected to do? The undefined space for professionalizable work in translational biomedicine. Minerva.

[2] Long JC, Hibbert P, Braithwaite J (2015). Structuring successful collaboration: a longitudinal social network analysis of a translational research network. Implement Sci.

[3] Logsdon MC, Kleiner C, Oster CA, Smith CD, Evans BB, Kempnich JM, et al. Description of Nurse Scientists in a Large Health Care System. Nurs Adm Q. 2017 20;41(3):266–74. 10.1097/NAQ.0000000000000237 28574897

[4] Ritchie MJ, Parker LE, Edlund CN, Kirchner JE (2017). Using implementation facilitation to foster clinical practice quality and adherence to evidence in challenged settings: a qualitative study. Bmc Health Serv Res.

[5] Barry M, de Groot E, Baggen Y, Smalbrugge M, Moolenaar N, Bartelink MLEL (2019). Understanding the broker role of clinician-scientists: a realist review on how they link research and practice. Acad Med J Assoc Am Med Coll..

[6] Thompson MR, Schwartz BD (2019). The role of the nurse scientist as a knowledge broker. J Nurs Scholarsh.

[7] Lander B (2016). Boundary-spanning in academic healthcare organisations. Res Policy.

[8] Bornbaum CC, Kornas K, Peirson L, Rosella LC (2015). Exploring the function and effectiveness of knowledge brokers as facilitators of knowledge translation in health-related settings: a systematic review and thematic analysis. Implement Sci.

[9] Wilson-Kovacs D, Hauskeller C (2012). The clinician-scientist: professional dynamics in clinical stem cell research. Sociol Health Illn.

[10] Hoeijmakers M, Harting J, Jansen M (2013). Academic collaborative centre limburg: a platform for knowledge transfer and exchange in public health policy, research and practice?. Health Policy.

[11] Adamsen L, Larsen K, Bjerregaard L, Madsen JK (2003). Danish research-active clinical nurses overcome barriers in research utilization. Scand J Caring Sci.

[12] Kluijtmans M, de Haan E, Akkerman S, van Tartwijk J (2017). Professional identity in clinician-scientists: brokers between care and science. Med Educ.

[13] Weggemans MM, Friesen F, Kluijtmans M, Prakken B, ten Cate O, Woods NN (2019). Critical gaps in understanding the clinician-scientist workforce: results of an international expert meeting. Acad Med.

[14] Kislov R, Wilson P, Boaden R (2017). The ‘dark side’ of knowledge brokering. J Health Serv Res Policy.

[15] Barry M, Kuijer W, Niewenhuis L, Scherpbier-de Haan N. Professional development arising from multiple-site workplace learning: boundary crossing between the education and clinical contexts. 2020. 10.1186/s12909-020-02225-y PMC7510255 32967683

[16] McKenney S, Reeves TC, Reeves TC (2018). Conducting educational design research.

[17] Parkinson S, Eatough V, Holmes J, Stapley E, Midgley N (2016). Framework analysis: a worked example of a study exploring young people’s experiences of depression. Qual Res Psychol.

[18] Tennant A (2007). Goal attainment scaling: current methodological challenges. Disabil Rehabil.

[19] Timonen H, Vuori J. Visibility of Work: How Digitalization Changes the Workplace. 10.

[20] Paton S, Hodgson D, Muzio D (2013). The price of corporate professionalisation: analysing the corporate capture of professions in the UK. New Technol Work Employ.

[21] Houle CO. Continuing Learning in the Professions. San Francisco: Jossey-Bass Publishers, 1980. Educ Forum. 1981;45(3):380–2.

[22] D’Ippolito B (2015). Conventional and less conventional mechanisms of professionalisation underpinning knowledge-intensive activities: the case of design in food industries. Ind Innov.

[23] Cervero RM (1992). Professional practice, learning, and continuing education: an integrated perspective. Int J Lifelong Educ.

[24] Akkerman SF, Bakker A (2011). Boundary crossing and boundary objects. Rev Educ Res.

